# Hydration Status and Fluid Needs of Division I Female Collegiate Athletes Exercising Indoors and Outdoors

**DOI:** 10.3390/sports7070155

**Published:** 2019-06-26

**Authors:** Stephanie Olzinski, Joshua Beaumont, Meynard Toledo, Amber Yudell, Carol S. Johnston, Floris C. Wardenaar

**Affiliations:** 1 College of Health Solutions, Arizona State University, Phoenix, AZ 85004, USA; 2 Sun Devil Athletics, Arizona State University, Tempe, AZ 85281, USA

**Keywords:** body weight change, fluid balance, urine specific gravity (USG), sweat rate, urine color, fluid recommendations

## Abstract

The purpose was to determine differences in acute and chronic hydration status in female student-athletes (*n* = 40) practicing in moderate, dry conditions (17–25 °C, 30–57% humidity) indoors and outdoors. Body weight and urine samples were recorded before and after exercise as well as fluid intake. Sweat rates expressed as median and interquartile range did not differ, but fluid intake was significantly higher during indoor (0.64 [0.50, 0.83] L/h) vs. outdoor conditions (0.51 [0.43, 0.63] L/h), *p* = 0.001. Fluid intake compensated for indoor sweat rate but not outdoors. When exercising indoors, 49% of the student-athletes reported urine specific gravity (USG) values >1.020, and 24% of the day after morning samples were scored ≥4 on the color chart rating. The percentages increased to 58% and 31%, respectively, when exercising outdoors (*p* > 0.05). Thus, fluid intake was higher indoors vs. outdoors but sweat rate did not differ among athletes. Yet, chronic hydration status was impaired in more than 50% of the student-athletes with a discrepancy between USG scores and urine color scores identifying underhydration. This suggest that 24-h fluid intake should be taken into account and that hydration protocols may need to be tailored individually based on urine USG values. Practice location (indoors vs. outdoors) may further complicate hydration protocols.

**Definitions:**

**Euhydration**—State of optimal total intracellular and extracellular body water content as regulated by the brain; 

**Hypohydration**—a state of water deficit with insufficient replacement of fluids caused by acute or chronic dehydration; 

**Dehydration**—the process of losing water from the body, during exercise, body water is most often lost through sweating; 

**Underhydration**—low water intake (consuming less than the reference values) or having high plasma osmolality in the absence of total body water deficit [1,2].

## 1. Introduction

Athletes are advised to consume enough fluid to stay in a well-hydrated state in order to maintain peak exercise performance [3]. The hindrance of performance when athletes are improperly hydrated is widely accepted, especially when the net body weight loss due to exercise-related sweating exceeds more than 2% of body mass [4,5]. Even slight levels of underhydration, as small as 1% of total body weight loss [4], cause a myriad of problems in athletes across all sport types [6,7,8,9]. A state of light dehydration, up to a severe water deficit, can cause many negative side effects including fatigue, cramps, heat exhaustion and heat stroke, decreased cognitive function, and in extreme cases loss of consciousness and death [4,6,7,8,9]. Fluid balance is achieved through the effects of antidiuretic hormones [10]. Loss of body water, resulting in dehydration, raises plasma osmolality levels which in turn stimulates the release of vasopressin, reducing the amount of water lost through the kidneys [6]. Despite this feedback loop, sweating induced by exercise may easily override the body’s fluid-saving mechanisms. If fluids are not properly replaced, impaired performance and exercise capacity may ensue [4,11,12]. Conversely, the atrial natriuretic peptide (ANP) is secreted in response to increased atrial blood volume, and promotes sodium, hence water, excretion [13]. With hotter temperatures, higher humidity, or a combination of both, athletes are at a greater risk of hypohydration from increased sweating, as insufficient fluid intake, both before and during practices/games, may occur due to limitations such as restricted drinking opportunities and play rules forbidding continuous fluid consumption during competition [3,14].

No single field-standard measurement to assess hydration status exists, therefore recommendation is to use at least two independent methods for the assessment of fluid balance and hydration status in the field [15]. Field measurements for acute and chronic hydration status are essential because they enable researchers, coaching staff, and athletes to evaluate hydration status and relate it to individual goals and performance. There is a substantial body of knowledge about fluid intake, fluid balance, and hydration status assessment in athletes [3,14,15,16,17]. A common, non-invasive and valid method of acute assessment includes monitoring changes in body weight before and after exercise which tracks acute changes in fluid balance [18,19]. For an athlete’s chronic hydration status urine specific gravity (USG) is easily measured [20] and can be used interchangeably with urine osmolality (Uosm) as these measurements are highly correlated (*r* = 0.995) [5,21]. Urine color (Uc) have been suggested as an accessible education tool for athletes as indicator of chronic hydration status [15] in assessing chronic hydration status in both adolescents and adults [22,23] in assessing hydration in both adolescents and adults. 

In areas above 37° latitude with annual average temperatures <15 °C [24], spring and summer sports will often train inside in winter time when it becomes too cold outside. Some indoor facilities can be climate-controlled to produce moderate-temperature conditions [25]. However, smaller indoor rooms, though shielded from direct sunlight exposure, can accumulate heat and relative humidity without proper ventilation, changing the environment during exercise and potentially affecting the athletes and their fluid requirements to compensate for increased sweating [26]. Increased sweating indoors has also been observed when athletes wear more clothing transitioning from hotter outdoor environments to cooler indoor environments [14]. In the Phoenix, Arizona metropolitan area, due to the high amount of sun hours, winters are relatively warm with average peak temperatures throughout fall and spring ranging from 19–31°C [27]. These temperatures are comparable to high spring and/or low summer temperatures in many other areas worldwide. Therefore, the Phoenix area lends itself optimally to assess any variances between fluid needs for sports exercising both indoors and outdoors under equal circumstances. 

The aim of this study was to determine potential differences in acute (e.g., sweat rate, body weight change, and fluid intake) and chronic hydration status (e.g., USG and Uc) in student-athletes exercising in different environmental conditions. To provide more insight into the needs for differentiation of fluid recommendations while exercising indoors versus outdoors and to explore the usefulness of urine color as potential tool for student-athletes to assess their hydration status. 

## 2. Materials and Methods

This quasi-experimental research study sought to determine the difference in fluid needs in a population of female student-athletes who exercised in both indoor and outdoor conditions with varying temperatures and levels of humidity. The total amount of student-athletes who consented to participating in the study were *n* = 47 (soccer *n* = 13; lacrosse *n* = 22; triathlon *n* = 12). The study was performed at the training facilities at Arizona State University (ASU) in Tempe, Arizona during March–April 2018, October 2018, and March–April 2019 for the three teams, respectively. The research protocol was reviewed and approved by the Institutional Review Board of Arizona State University: STUDY00007260. All student-athletes signed the informed consent before the start of the study. 

### 2.1. Approach

The student-athletes were observed during one indoor and one outdoor practice, and the training load, duration (~1.5–3 h), and temperature were consistent between study days. At the start of the study, the student-athletes provided information about their drinking preference (68% preferred sports drink with zero kcals and water and 32% preferred water only) during exercise. The student-athletes were randomly assigned a study ID number which identified the student-athlete, their drinking bottles, urine sample cups, and performance scores during exercise. 

Soccer practices included a warm up, running drill, ball passing, and scrimmages; lacrosse practices included warm up, running drill and conditioning, ball passing, and real play exercises. At the outdoor day the lacrosse team practiced indoors for 60 min and outdoors for 120 min. Triathlon performed cycling on stationary bicycles indoors at 2–3-min intervals combined with relative rest, or mobile bicycles outdoors having the same type of exercise as indoors. 

Before and after practice, each student-athlete was asked to provide an all-out urine sample, a semi-nude body weight in dry spandex, a sports bra, and socks. Drinking bottles were provided ad libitum and were measured pre- and post-practice. Other data collected during each study day included total minutes for exercise, exercise performed, distance covered and-or heart rate (including breathing rate and activity tracking) to estimate energy expenditure and environmental measurements. At the end of each practice session, all student-athletes were given a small urine sample cup to take home to deliver a mid-stream next morning sample between 6–9 am. 

### 2.2. Measurements

#### 2.2.1. Fluid and Food Intake

Student-athletes were provided with drinking bottles (e.g., zero-kcal sports drink and water or water alone) at the start of practice and were instructed to self-identify if they wanted a new bottle during practice. The bottles were filled with water or zero-calorie sports drink based on preference as identified in the screener questionnaire. The bottle weight was measured twice using a precision scale (PT 1400, Sartorius AG, GÖttingen, Germany) before and after practice. All student-athletes were encouraged to drink ad libitum throughout practices. They were asked to not spit out the drink or to use it to rinse their mouths. Any snacks that they consumed within 15 min before and after practice were also weighed in duplicate (PT 1400, Sartorius AG) and recorded in grams to be included in the final weight calculation (weight change was calculated using pre- and post-weight measurements minus the weight from food intake).

#### 2.2.2. Urine Collection and Body Weight Difference

To assess acute hydration status all student-athletes were provided with the opportunity to use a mobile field bathroom facility during practice. Urine was weighed to determine weight loss that was not due to sweat loss. No fecal matter was collected at any time point. Directly after the student-athletes delivered their pre- and post-exercise urine sample, body weight was recorded in duplicate (896 Digital Scale, Seca, Seca GmbH & Co. KG. Hamburg, Germany). At one of the measurement days the student-athletes height was measured as well (Portable Stadiometer, Seca, Seca GmbH & Co. KG. Hamburg, Germany). The triathletes performed two training sessions (swimming and cycling), but only the data collection for the bike training was included as this practice was performed indoors and outdoors. 

#### 2.2.3. Urine Specific Gravity and Urine Color

USG and Uc were determined to assess each athlete’s chronic hydration status. Both the pre-practice urine sample and the day after urine sample were analyzed for USG. For each urine sample 20 mL of urine was transferred into 30 mL test tubes for same-day analysis of USG after the samples were brought to 20 °C ± 0.5 °C (Traceable thermometer, Fisher Scientific, with ±0.2 °C accuracy) by standing for 2–3 h at the onsite laboratory facility. USG was measured using a digital handheld refractometer (Pen–Urine S.G., Atago, Tokyo, Japan) per manufacturer’s instructions. Duplicate measurements were averaged, and if there was >0.005 variation between the two measurements, a third measurement was performed and the median was used.

Uc was tested after the samples of all teams were collected and had been frozen at −20 °C for at least one week. All urine samples were brought back to 20 °C using a warm water bath ranging in temperature from 30–40 °C. All samples were inverted twice directly before measurements of Uc to redistribute particles that might have precipitated. The test tubes (30 mL freestanding centrifuges tubes with green lids, Evergreen Scientifics, Vernon, California, USA) were placed in a urine color-scoring box that was developed by this lab with the intent to standardize the scoring of urine samples. The box had three slots at the back where samples in test tubes could be placed on top of small LED lights (each slot contained one flashlight with 6 LEDs providing 20 Lumens, Ozark Trail, Ozark, AR, USA). The inside of the box was painted black to eliminate reflections of the light source. Directly behind the sample was a plain white background and the box was covered with a lid during the testing of each sample. At the user’s end three holes were present that allowed scoring of each sample. For the purpose of this study, only one sample was placed at the central spot of the box and the other two windows were sealed. Two research team members scored the samples separately using the 8-scale urine color chart developed by Armstrong [15]. After this, both research team members agreed upon which color from the chart the urine sample most closely matched. 

#### 2.2.4. Heart Rate, Breathing Rate and Activity

For the estimation of energy expenditure, heart rate (HR), breathing rate (BR), and activity were measured continuously until the end of exercise for each student-athlete using an elastic chest strap tracker (Bioharness-3, Zephyr Technology, Medtronic, Boulder, CO, USA). This device includes a HR monitor and a 3-axis accelerometer with a sensor to measure BR. Mean HR and skin temperature were calculated as averages, excluding the values measured directly before the start or after the end of each training session.

#### 2.2.5. Temperature, Humidity, and Wet Bulb Globe Temperature

During each practice, the environmental temperature, relative humidity, and wet bulb globe temperature (WBGT) were measured in 10-min intervals using portable climate monitoring device with a wind vane (Kestrel 5400 Heat Stress Tracker, Nielsen-Kellerman, Boothwyn, PA, USA), which were positioned on a tripod at chest height in the sun when practicing outdoors, or indoors. The WBGT was calculated using the formula: WBGT = 0.1 (Tdry bulb) + 0.7 (Twet bulb) + 0.2 (Tglobe) [15]. 

### 2.3. Calculations

Sweat rate was calculated as the amount of fluid lost per hour (L/h) in the form of sweat: (Pre-exercise BM (kg) − post-exercise BM (kg)) + (fluid ingested (L) − urine output (L))/(protocol duration (min) × 60) [28]. Energy expenditure was estimated in metabolic equivalents (METs) each minute based upon a linear regression approach derived from the 3-axis accelerometer, HR, and BR inputs. The following formula was used: METs = −1.1644 + 7*HR + 5.8985*Activity Score + 3*BR [29]. METs were calculated towards energy expenditure (kcal) using the formula: METs*body weight (kg)*hours of exercise [30].

### 2.4. Statistical Analysis

Descriptive statistics and differences between groups were calculated (SPSS 21 for Windows, 2010, IBM, Armonk, NY, USA). Descriptive data (baseline characteristics) are presented as mean ± SD, and differences between means were assessed using the one-way analysis of variance test (*p* ≤ 0.05); however, outcome data were not normally distributed and were thus reported as median and interquartile range (IQR). The environmental values were the same for all student-athletes within each team for separate testing days. A Wilcoxon Signed Rank test determined differences between indoor and outdoor conditions. Significance was set at *p* < 0.0167 which is the Bonferroni correction using the critical *p*-value (0.05) divided by 3 comparisons. Kruskal-Wallis tests determined differences between all three sports—soccer, lacrosse, and triathlon (*p* ≤ 0.05), and Mann–Whitney U tests determined differences between two sports compared against each other (*p* ≤ 0.05). Relationships between variables were assessed using Spearman’s rank correlation coefficient and 95 % CI were calculated using Fisher’s Z transformation, nominal data were compared using the chi-square independence test.

## 3. Results

Complete indoor and outdoor data were obtained for 40 student-athletes and these data were used for all analyses. Baseline characteristics for the student-athletes did not vary by sport (Table 1).

### 3.1. Training Conditions

The ambient temperature (23.3 °C vs. 20.5 °C) and WBGT (18.0 °C vs. 17.5 °C) were significantly different between indoor and outdoor study days with higher values produced indoors (*p* < 0.001) as shown in Table 2. Differences were also found for ambient temperature, WBGT, and humidity between soccer and lacrosse, soccer and triathlon, and lacrosse and triathlon for these variables on both indoor and outdoor days (*p* < 0.001), indicating that measurements were collected during a range of moderate temperatures indoors (19–25 °C) and outdoors (17–23 °C). 

No differences were seen for the combined group of athletes between indoor and outdoor practices for heart rate: 137 [129, 150] bpm. vs. 142 [130, 158] bpm., *p* = 0.199. Additionally no difference between indoor and outdoor practices was seen based on calculated METs: 5.1 [4.2, 6.0] vs. 5.2 [4.4, 7.5], with *p* = 0.157, and the average estimated energy expenditure estimated (EEe) per hour did not show a difference between environmental conditions (318 [269, 389] kcal/h vs. 305 [272, 425] kcal/h, *p* = 0.183). Significant differences between indoors and outdoors were found for heart rate for soccer and lacrosse (*p* = 0.007), and between METs and EEe for the combined group of athletes (*p* ≤ 0.001, *p* ≤ 0.001) due to natural differences of the type and intensity of practices. 

### 3.2. Acute Changes in Hydration Status

#### 3.2.1. Body Weight Difference (kg and %)

Significance was found for body weight change (−0.05 [−0.38, 0.18] kg vs. −0.02 [−0.48, −0.10] kg, *p* = 0.004) and percent weight change (−0.07 [−0.59, 0.25] % vs. −0.32 [−0.75, −0.15] %, *p* = 0.003) between indoor and outdoor days. No additional differences were found between any sports (*p* > 0.05).

#### 3.2.2. Fluid Intake and Urine Output

**Figure 1:**
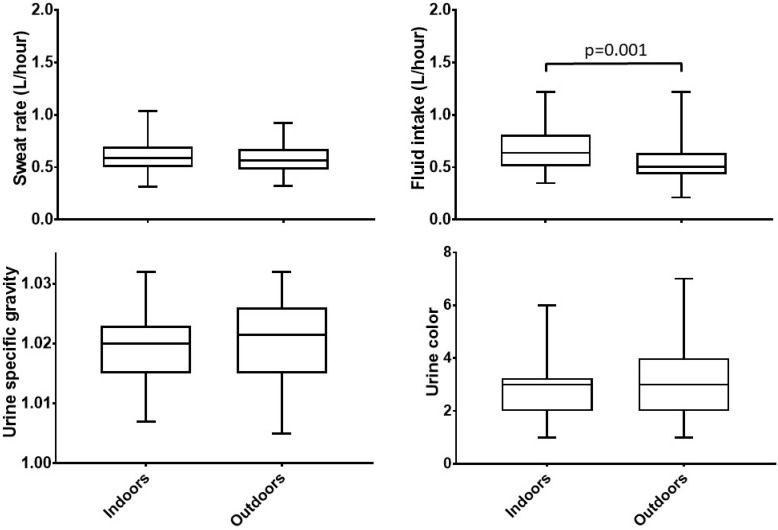
Indoor and outdoor comparisons for sweat rate (L/h), fluid intake (L/h), urine specific gravity (USG), and urine color (Uc).

Fluid intake was significantly higher during indoor (0.64 [0.50, 0.83] L/h) vs. outdoor conditions (0.51 [0.43, 0.63] L/h), indicated by *p* = 0.001 as shown in Figure 1. Fluid intake differed between sports indoors with lacrosse having the lowest intake rate (0.58 [0.45, 0.66] L/h) vs. other sports (soccer and triathlon, 0.76 [0.53, 0.94] and 0.84 [0.60, 0.89] *p* = 0.043) (Table 3). 

No differences were observed for total urine output between indoor and outdoor study days (*p* = 0.317), with the results showing a modest output of 0.08 (0.03, 0.21) L and 0.11 (0.06, 0.14) L per practice session. 

#### 3.2.3. Sweat Rate

Sweat rate was not significantly different between indoors (0.59 [0.50, 0.70] L/h) and outdoors (0.56 [0.48, 0.68] L/h) with *p* = 0.168 and no differences were found between sports (*p* > 0.05). The fluid intake for the combined group of athletes compensated for the sweat rate indoors, but not outdoors. Fluid intake was correlated to sweat rate both indoors (r = 0.452, *p* = 0.003) and outdoors (r = 0.517, *p* < 0.001).

### 3.3. Chronic Hydration Status

#### 3.3.1. Urine Specific Gravity

**Figure 2:**
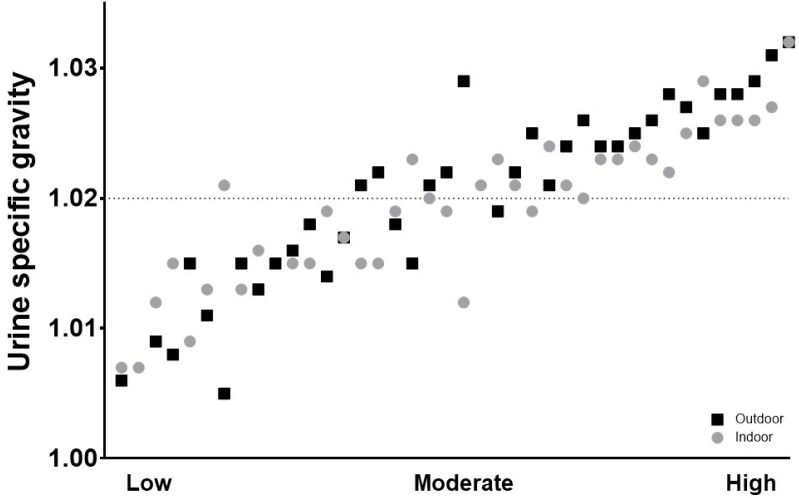
Day after USG values for combined sports. Indoor and outdoor values plotted together for each student-athlete and organized from low-high USG. Indoor and outdoor USG values were significantly correlated, *r* = 0.665 [95% CI 0.45–0.81, *p* < 0.001].

In Figure 2, day after USG values were plotted for all athletes for both indoor and outdoor conditions. The values ranged from 1.007–1.032 indoors and 1.005–1.032 outdoors. The USG values were close in range between indoor and outdoor conditions among all athletes (r = 0.665, *p* < 0.001). When exercising indoors, 49% of the student-athletes reported USG values above the suggested cutoff value for under-hydration of 1.020, and the percentage increased to 58% when exercising outdoors (*p* > 0.05).

No differences existed between total indoor and outdoor conditions for either pre-practice USG values (1.018 [1.008, 1.023] vs. 1.022 [1.012, 1.026], *p* = 0.058), or day after USG values (1.020 [1.015, 1.023] vs. 1.022 [1.015, 1.026], *p* = 0.230). No significant differences were found between sports for indoor or outdoor practices (*p* > 0.05) (Figure 1). 

When the combined group was split into low and high USG values, as approximately 50% of student-athletes tended to have elevated USG values >1.020, no differences were found between the groups in their body weight difference, percentage body weight, fluid intake, urine output, or sweat rate surrounding exercise either indoors or outdoors.

Splitting the group based on type of fluid consumed (e.g., water or a combination of water and a zero kcal sports drink) revealed no differences for acute hydration variables, but a significant mean difference was seen between both groups for day after USG values (median value for water 1.024 vs. 1.020 for the combination of water and sports drink, *p* ≤ 0.026), this was also reflected in a higher percentage water consumers above the 1.020 cutoff then the student-athletes drinking both water and sports drink (71% vs. 0.49%).

#### 3.3.2. Urine Color

Based upon the urine color chart, a score of 1–3 indicates euhydration, while a score of 4–8 represents a state of light to severe hypo- or underhydration. No differences were found for urine color between indoors and outdoors (3.0 [2.0, 3.3] vs. 3.0 [2.0, 4.0], *p* = 1.000). For indoor conditions, Uc scores for lacrosse and triathlon (3.0 [2.0, 5.0] vs. 2.0 [1.8, 2.3]) differed significantly (*p* = 0.020). When exercising indoors, 24% of the day after morning samples were scored ≥4 on the color chart rating and 31% of the samples after the outdoor practice scored ≥4 for urine color (*p* > 0.05). Uc was strong correlated with USG values indoor with r: 0.654 [95% CI 0.41–0.80, *p* < 0.001] and outdoor with *r =* 0.600 [95% CI 0.35–0.77, *p* < 0.001].

## 4. Discussion

This study adds to our knowledge reporting fluid and hydration behavior of female athletes from a selection of various sports, including both team sports (internationally-based for soccer, American-based for lacrosse), and an endurance sport (triathlon). Studies have focused on athletes exercising in a variety of environments [16,31,32], but not making a direct comparison between differences in fluid needs or hydration status indoors and outdoors. The major findings from this study were that fluid intake was higher on indoor days, suggesting increased intake stimulation from indoor environments where environmental temperature and/or humidity are slightly elevated, and, although not statistically significant, a higher percentage of athletes scored in the dehydration range (as indicated by both USG and color) following practice outdoors. Moreover, as a group, athletes’ fluid intakes compensated for sweat rates during the indoor practice only. Hence, drinking may need to be encouraged particularly when practicing outdoors. About half of the student-athletes reported USG values >1.020 the day after practice with a more profound effect after an outdoor practice, based on urine color assessment a smaller population was identified as being ‘dehydrated’. 

Research has not found a definite connection between higher humidity and consistently greater fluid intakes during exercise [33], but student-athletes in the current study consumed significantly more fluid during exercise indoors. The triathletes were experiencing the highest humidity due to the fact that their indoor training was completed in a small, poorly-ventilated room whereas the others exercised in a large pressurized inflatable dome. In this instance, the results are not indicative of a clear cause of thirst response and fluid intake as either humidity or temperature could be a factor. However, with increasing levels of humidity, drinking to thirst may not suffice as the results showed that 50% of athletes were already underhydrated based on USG measurements in pre-practice urine samples. Earlier studies of fluid intakes for marathon runners in varying levels of ambient temperature and humidity in different areas of the world showed mixed results. While some runners had increased fluid intakes with higher levels of relative humidity, others had decreased fluid intakes in similar temperatures and humidity [33]. Fluid intake has also been linked to the length and amount of formal opportunities to drink for team sports [32]. The present population of student-athletes did not become dehydrated during practice, probably because of the ample availability of fluid and opportunities to drink. Fluid availability was ad libitum and on average every 15 min there was a drink break on both testing days. Although sweat rates are highly individual, athletes are generally recommended to consume 0.4–0.8 L of fluid per hour of exercise [9] which was met on both indoor and outdoor days by the athletes. This group also met individual needs, in most cases staying within the recommended 1% body weight change range. Sweat rate dictates fluid needs during exercise and some differences in sweat rates were found between sports. Herein, sweat rates were significantly correlated to intake rates both indoors and outdoors.

As absolute output was collected and not corrected for practice time, urine output differed between teams. These natural differences were seen between groups both during indoor and outdoor practices due to the total duration of practice. Sweat rates were comparable between days and sports and an increase in sweat rate was unlikely to influence urine excretion. Through high sweat rates, the body saves fluid through the hormonal feedback loop, reducing the kidney output [6]. The median outputs found in this study were low compared to other studies, for example, athletes exercising in climate-controlled chambers with higher temperatures and humidity (32.0 ± 0.4 °C and 53.8 ± 5.2% humidity) had higher outputs of urine (0.59–0.89 L), showing a similar connection between humidity and urine output as was seen with humidity and fluid intake [34]. Broad and colleagues determined that in warmer temperatures during summer training (25 °C), there was increased sweat rate, increased fluid intake, and more instances of hypohydration compared to the winter training season (9 °C) for junior elite male soccer players [14,35]. In the current study, total and individual team fluid intake covered losses from sweating for indoors but not outdoors, however sweat rates were moderate and equal between indoors and outdoors. Therefore fluid recommendations may need to consider the practice environment (indoors or outdoors) as well as the drinking habits of individual athletes and the duration or intensity of the exercise.

Chronic dehydration may be a signifying potential risk factor for dehydration regardless of practice environment. If student-athletes do not drink enough during the rest of the day, fluid consumption during practices may be a very important mechanism for student-athletes to compensate for these lower fluid intakes. As was seen in this study, no differences were found in sweat rate or body weight change in the student-athletes participating in indoor and outdoor conditions. Although student-athletes had significantly greater fluid intakes indoors compared to outdoors, both conditions revealed that chronic hydration markers were altered as evidenced by elevated day after USG values. Therefore, again in moderate conditions with the opportunities that these student-athletes had (e.g., ample drinking opportunities and ad libitum availability of drinks), their intake was probably sufficient during practice. Between the pre-practice and day after practice urine samples surrounding exercise, there were no differences between indoor and outdoor conditions, but more than half of the student-athletes reported the day after practice USG values >1.020 both indoors and outdoors. Other studies also reported substantial numbers with high USG levels ranging from 31.9% and 43.6% on the day of practice [36] with up to 89% of participants being dehydrated the day after exercise [20]. For soccer specifically, a Chilean study found that out of 156 professional players from all regions of the country, 90% were significantly or severely dehydrated prior to commencing exercise [37]. As shown in this study, the group consuming water during practice reported higher morning after USG values. There were no absolute differences in fluid consumption between water drinkers and the ones drinking a combination water and sports drink and therefore the contribution of electrolytes as part of the beverage influencing fluid balance was limited. It could be that student-athletes that consumed sports drink were more aware than those consuming water of the importance of proper hydration or they may have been trying harder to maintain a good fluid balance. Regardless of the difference between these groups based on the type of fluid consumed, there was still a substantial portion of student-athletes in the sports drink group that reported high USG values and when all student-athletes were combined the prevalence of USG values >1.020 was comparable to earlier reports [20,36]. Kavouras recently suggested that the term underhydration should be used when no signs of a deficit of total body water in combination with elevated urinary markers are present [2]. Since 50% of the student-athletes were found to be underhydrated during this study, while fluid intake during practice was sufficient on a group level, this underhydration may not have been related to their fluid intake as body weight change was almost zero. Thus, the student-athletes likely did not drink sufficient amounts of fluid throughout the rest of the day outside of practice times. 

The interest of adding urine color as an additional field test to this study was to define the potential future use of urine color chart scoring by athletes to assess their own chronic hydration status. We found correlation scores that were comparable with earlier reported correlations (*r*: 0.60–0.80) [22,23]. Taking into account that the reporting of athletes may be different from that of trained research personnel, not much is known about the quality of self-reporting of urine color by athletes. As far as we know, only one study that assessed self-reporting in a young group of soccer players, showing that an eight-point color chart is equally valid between research assessment and self-assessment [23]. The scoring of urine color by our research team in this study showed a difference in classification of student-athletes being hydrated vs. underhydrated. In comparison to USG analysis, only half of the underhydrated student-athletes would be signaled being underhydrated when using the color chart. As study participants tend to score their own urine color at least one color shade lower in comparison to the actual color (i.e., the color defined by trained research personnel) [23], based on unpublished pilot data from our lab, we expect that the method may need revision to be useful as an actual field evaluation test supporting self-hydration assessment and decision making in athletes. 

As a result of a field approach the practice workload was not standardized. Although the teams performed different exercises during practices, the effort they were delivering was comparable for indoors and outdoors based upon the similarities of the type of training program, HR, METs, and EEe. No differences were found between indoor and outdoor conditions for any of these activity factors. The temperature ranges while testing were all within the limits of being moderate which allowed for a correct comparison between the three sport teams that were tested. Finally, all methods for collecting, measuring, and storing data were the same for each team, and thus standardization of testing was achieved. Another limitation of this study was that fluid intake behavior may have been influenced as athletes may adjust their behaviors when participating in research, the student-athletes knew they were being measured and observed which may have influenced their drinking behavior [25]. On the other hand, coaches kept practices close to normal and in real time drinks are available ad libitum as well and all coaches said that this amount of drinking breaks was part of the regular practice approach.

In conclusion, this research showed that in a dry environment under moderate temperature conditions there was no serious concern for acute dehydration during regular practice as student-athletes stayed within 1% of body weight loss during both indoor and outdoor practice. Although, sweat rate did not differ during indoor vs. outdoor practices, fluid intake was higher during indoor practice vs. outdoor practice, and fluid intake compensated for sweat rate during indoor practice only. The assessment of chronic hydration status based on USG values, independent of practice location and environment, identified half of the student-athletes as being underhydrated. These data suggest that 24-h fluid intake should be taken into account and that hydration protocols may need to be tailored individually based on urine USG values. Practice location (indoors vs. outdoors) may further complicate hydration protocols. Additional research is necessary to further detail hydration needs and at home assessment of hydration status (e.g., including urine color assessment) when exercising indoors vs. outdoors to fine-tune hydration protocols for collegiate student-athletes. 

## Figures and Tables

**Table 1:** Baseline characteristics of student athletes by sport (mean ± SD).

	Total (*n* = 40)	Soccer (*n* = 10)	Lacrosse (*n* = 19)	Triathlon (*n* = 11)	*p*-Value
Age, y	19.9 ± 1.4	19.8 ± 1.2	18.8 ± 0.76	20.0 ± 1.6	*0.055*
Height, cm	167.7 ± 7.1	171.6 ± 6.0	167.9 ± 4.6	163.7 ± 9.6	*0.083*
Weight, kg	62.6 ± 5.8	64.0 ± 7.2	63.5 ± 4.2	59.6 ± 6.3	*0.247*
BMI, kg/m^2^	22.3 ± 2.0	21.7 ± 1.6	22.6 ± 1.3	22.4 ± 3.2	*0.765*

*p*-Value represents one-way ANOVA; there were no differences between groups at baseline.

**Table 2:** Environmental Conditions and Exercise Output during Indoor and Outdoor conditions for Total and Individual sports (median and interquartile range (IQR)).

		Total		Sports			
				Soccer	Lacrosse	Triathlon	*p*-Value
**Ambient temp (°C)**	Indoor	23.3 (20.4, 24.7)		19.4 ^ab^	23.3 ^ac^	24.7 ^bc^	*<0.001*
	Outdoor	20.5 (17.4, 23.3)		16.4 ^ab^	23.3 ^ac^	20.5 ^bc^	*<0.001*
	*p*-value	*<0.001*		*0.02*	*1.000*	*0.001*	
**Relative humidity (%)**	Indoor	35.3 (29.8, 57.0)		28.0 ^ab^	35.3 ^ac^	57.0 ^bc^	*<0.001*
	Outdoor	38.0 (34.3, 38.4)		33.0 ^ab^	38.4 ^ac^	38.0 ^bc^	*<0.001*
	*p*-value	*0.732*		*0.02*	*0.00*	*0.01*	
**WBGT (°C)**	Indoor	18.0 (14.1, 25.6)		12.8 ^ab^	25.6 ^ac^	18.0 ^bc^	*<0.001*
	Outdoor	17.5 (14.6, 30.8)		13.6 ^ab^	30.8 ^ac^	17.5 ^bc^	*<0.001*
	*p*-value	*<0.001*		*0.02*	*0.00*	*0.01*	
**Heart rate (bpm)**	Indoor	137 (129, 150)		156 (140, 165) ^ab^	131 (122, 137) ^a^	137 (134, 139) ^b^	*0.008*
	Outdoor	142 (130, 158)		163 (156, 170) ^ab^	130 (120, 140) ^a^	135 (132, 145) ^b^	*<0.001*
	*p*-value	*0.199*		*0.114*	*0.265*	*0.683*	
**METs**	Indoor	5.1 (4.2, 6.0)		7.8 (6.0, 7.9) ^ab^	5.2 (4.7, 5.4) ^ac^	4.1 (4.1, 4.2) ^bc^	*<0.001*
	Outdoor	5.2 (4.4, 7.5)		8.0 (7.5, 8.4) ^ab^	5.3 (4.8, 5.5) ^ac^	4.1 (4.0, 4.4) ^bc^	*<0.001*
	*p*-value	*0.157*		*0.173*	*0.166*	*0.681*	
**Energy expenditure estimated (EEe, kcal/h)**	Indoor	318 (269, 389)		509 (459, 552) ^ab^	318 (318, 338) ^ac^	242 (219, 265) ^bc^	*<0.001*
	Outdoor	305 (272, 425)		492 (473, 585) ^ab^	305 (305, 340) ^ac^	236 (209, 279) ^bc^	*<0.001*
	*p*-value	*0.183*		*0.445*	*0.012*	*0.534*	

Wilcoxon signed rank test determined differences between indoor and outdoor conditions. Kruskall–Wallis determined differences between all three sports—soccer, lacrosse, and triathlon—and then Mann–Whitney U tests determined differences between two sports compared against each other. Different superscripts signify significant differences between sports based upon the Mann–Whitney U test with *p*-value ≤ 0.05: ^a^ Soccer and lacrosse. ^b^ Soccer and triathlon. ^c^ Lacrosse and triathlon.

**Table 3:** Bodyweight difference (kg and %) and hydration indicators during indoor and outdoor conditions for total and individual sports (median and IQR).

		Total		Sports			
				Soccer	Lacrosse	Triathlon	*p*-Value
**Body weight difference (kg)**	Indoor	−0.05 (−0.38, 0.18)		0.10 (−0.15, 0.20)	−0.10 (−0.40, 0.00)	−0.10 (−0.50, 0.30)	*0.284*
	Outdoor	−0.20 (−0.48, −0.10)		−0.20 (−0.35, −0.10)	−0.20 (−0.50, 0.00)	−0.10 (−0.50, 0.00)	*0.930*
	*p*-value	*0.004*		*0.059*	*0.022*	*0.384*	
**Percentage weight change**	Indoor	−0.07 (−0.59, 0.25)		0.18 (−0.22, 0.31)	−0.15 (−0.63, 0.00)	−0.18 (−0.72, 0.52)	0.209
	Outdoor	−0.32 (−0.75, −0.15)		−0.29 (−0.55, −0.17)	−0.34 (−0.78, 0.00)	−0.18 (−0.84, 0.00)	0.986
	*p*-value	*0.003*		*0.047*	*0.022*	*0.386*	
**Sweat rate (L/h)**	Indoor	0.59 (0.50, 0.70)		0.69 (0.43, 0.86)	0.57 (0.51, 0.67)	0.58 (0.50, 0.94)	*0.428*
	Outdoor	0.56 (0.48, 0.68)		0.61 (0.51, 0.86)	0.57 (0.49, 0.68)	0.54 (0.42, 0.63)	*0.274*
	*p*-value	*0.168*		*0.541*	*0.920*	*0.110*	
**Fluid intake (L/h)**	Indoor	0.64 (0.50, 0.83)		0.76 (0.53, 0.94) ^a^	0.58 (0.45, 0.66) ^ac^	0.84 (0.60, 0.89) ^c^	*0.043*
	Outdoor	0.51 (0.43, 0.63)		0.49 (0.42, 0.67)	0.54 (0.46, 0.66)	0.50 (0.37, 0.63)	*0.854*
	*p*-value	*0.001*		*0.059*	*0.376*	*0.004*	
**Urine output (L)**	Indoor	0.08 (0.03, 0.21)		0.03 (0.02, 0.12) ^b^	0.08 (0.03, 0.13) ^c^	0.14 (0.08, 0.33) ^cb^	*0.020*
	Outdoor	0.11 (0.06, 0.15)		0.03 (0.03, 0.06) ^ab^	0.11 (0.09, 0.15) ^a^	0.14 (0.13, 0.31) ^b^	*<0.001*
	*p*-value	*0.317*		*0.838*	*0.064*	*0.859*	
**USG pre-practice**	Indoor	1.018 (1.008, 1.023)		1.025 (1.023, 1.028) ^ab^	1.011 (1.006, 1.021) ^a^	1.013 (1.008, 1.020) ^b^	*<0.001*
	Outdoor	1.022 (1.012, 1.026)		1.023 (1.017, 1.028)	1.023 (1.011, 1.027)	1.017 (1.006, 1.024)	*0.288*
	*p*-value	*0.058*		*0.386*	*0.017*	*0.236*	
**USG day after**	Indoor	1.020 (1.015, 1.023)		1.022 (1.018, 1.026)	1.021 (1.017, 1.023)	1.015 (1.012, 1.021)	*0.063*
	Outdoor	1.022 (1.015, 1.026)		1.023 (1.015, 1.028)	1.022 (1.017, 1.026)	1.017 (1.009, 1.025)	*0.246*
	*p*-value	*0.230*		*0.440*	*0.233*	*0.838*	
**Urine color day after (Uc)**	Indoor	3.0 (2.0, 3.3)		2.5 (2.0, 3.0)	3.0 (2.0, 5.0) ^c^	2.0 (1.8, 2.3) ^c^	*0.040*
	Outdoor	3.0 (2.0, 4.0)		2.0 (2.0, 4.0)	3.0 (3.0, 4.0)	2.0 (1.0, 3.3)	*0.114*
	*p*-value	*1.000*		*0.589*	*0.904*	*0.414*	

Wilcoxon signed rank test determined differences between indoor and outdoor conditions. Kruskall–Wallis determined differences between all three sports—soccer, lacrosse, and triathlon—and then Mann–Whitney U tests determined differences between two sports compared against each other. Different superscripts signify significant differences between sports based upon the Mann–Whitney U test with *p*-value ≤ 0.05: ^a^ Soccer and lacrosse. ^b^ Soccer and triathlon. ^c^ Lacrosse and triathlon.
